# Localized network damage related to white matter hyperintensities is linked to worse outcome after severe stroke

**DOI:** 10.1186/s42466-025-00416-w

**Published:** 2025-08-19

**Authors:** Samuel C. Olszówka, Benedikt M. Frey, Jan F. Feldheim, Lukas Frontzkowski, Paweł P. Wróbel, Winifried Backhaus, Focko L. Higgen, Hanna Braaß, Silke Wolf, Chi-un Choe, Marlene Bönstrup, Bastian Cheng, Götz Thomalla, Philipp J. Koch, Fanny Quandt, Christian Gerloff, Robert Schulz

**Affiliations:** 1 Department of Neurology, University Medical Center Hamburg-Eppendorf, Martinistraße 52, 20246 Hamburg, Germany; 2 Department of Neurology, University Medical Center Frankfurt, Frankfurt, Germany; 3 Department of Neurology, Charite University Medical Center Berlin, Berlin, Germany

**Keywords:** Cerebral small vessel disease, WMH, Brain reserve, Functional outcome, Rehabilitation

## Abstract

White matter hyperintensities of presumed vascular origin (WMH) are associated with various clinical sequelae. In stroke patients, the total WMH burden is linked to recurrent cerebrovascular events and worse clinical outcomes. As WMH also affect the integrity of large-scale structural brain networks, we hypothesize that the extent of WMH-related network damage carries relevant information to explain outcome variability in addition to global WMH volume. Clinical and structural brain imaging data of 33 severely affected acute stroke patients were analyzed from two independent cohorts. Imaging data were acquired within the first two weeks after stroke. WMH-related localized and global network damage was derived. WMH network effects were differentially assessed for total, periventricular (pWMH), and deep WMH (dWMH). Using ordinal logistic regression analyses, network damage was associated with functional outcome at follow-up after three to six months. WMH were linked to a significant disconnection of multiple cortical and subcortical brain regions. Global and localized pWMH-related network damage affecting distinct brain regions of both hemispheres were independently associated with a worse outcome after adjustment for baseline symptom burden, age, brain infarct volume, and total WMH volume. Total and dWMH-related network disturbances did not show similar associations. This study indicates that pWMH-related network damage affecting specific brain regions is linked to functional outcome in acute stroke patients. It underscores the potential significance of pre-existing WMH-related network damage as a crucial factor in comprehending outcome variability after severe stroke.

**Supplementary Information:**

The online version contains supplementary material available at 10.1186/s42466-025-00416-w.

## Introduction

White matter hyperintensities of presumed vascular origin (WMH) are a manifestation of cerebral small vessel disease (cSVD) and are associated with various clinical sequelae such as cognitive decline, dementia, depression as well as disturbances of gait and balance [1, 2, 3]. Based on their distance to the inner ventricle system, WMH can be divided into deep WMH (dWMH) and periventricular WMH (pWMH), with the two forms being related to different risk factors and neurological impairment [4]. WMH are also a common finding in magnetic resonance imaging (MRI) of ischemic stroke patients, and 25% of ischemic strokes can be attributed to the presence of cSVD [5]. WMH burden is linked to higher probabilities of first-ever [1] or recurrent strokes [6] as well as a worse outcome after stroke [6, 7, 8].

The factors driving the association between WMH and outcome after stroke remain largely unknown. From a clinical perspective, WMH might reflect the systemic burden of vascular risk factors, even though the association of WMH and clinical outcome remained stable in adjusted meta-analyses [6, 8]. WMH may also exert a behavioral influence on stroke recovery [9, 10], for instance, through cognitive dysfunctions related to WMH, which reduce patients’ adherence to and participation in neurorehabilitative treatment [8]. However, from a systems neuroscience perspective, WMH may impact recovery trajectories by affecting large-scale structural and functional brain networks [11, 12] as numerous studies have consistently reported that the state of structural brain networks, quantified early after acute stroke, can inform about subsequent recovery [13, 14, 15], and that the location of WMH influences stroke severity [16]. WMH-related network damage could serve as a surrogate for pre-existing disturbances in brain networks, and it could be a novel approach to better understand the underlying mechanisms of brain reserve capacity [17] and outcome variability after stroke, as recently evidenced for cortical and cerebellar brain anatomy [18, 19]. Recently, one study showed that incorporating WMH-related network disconnections improved the prediction of non-verbal cognitive deficits in poststroke aphasia [20]. Building on these findings, the present study sought to answer whether the extent of WMH-related network damage, quantified early after severe ischemic stroke, can also improve prospective outcome modeling. Generally, stroke patients with mild impairment show recovery proportional to their initial impairment. In patients with severe stroke deficit, however, 30% of patients do not show such proportional recovery [21]. Hence, variability in outcome in this subset of patients is more considerable, and driving factors are still up for debate. To this end, clinical and structural brain imaging data of 33 severely affected acute stroke patients were analyzed [22, 23]. WMH-related localized and global network damage was calculated for multiple cortical and subcortical brain regions. Network damage was associated with functional outcome, operationalized by the modified Rankin Scale (mRS) at follow-up.

## Methods

### Participants

**Fig. 1:**
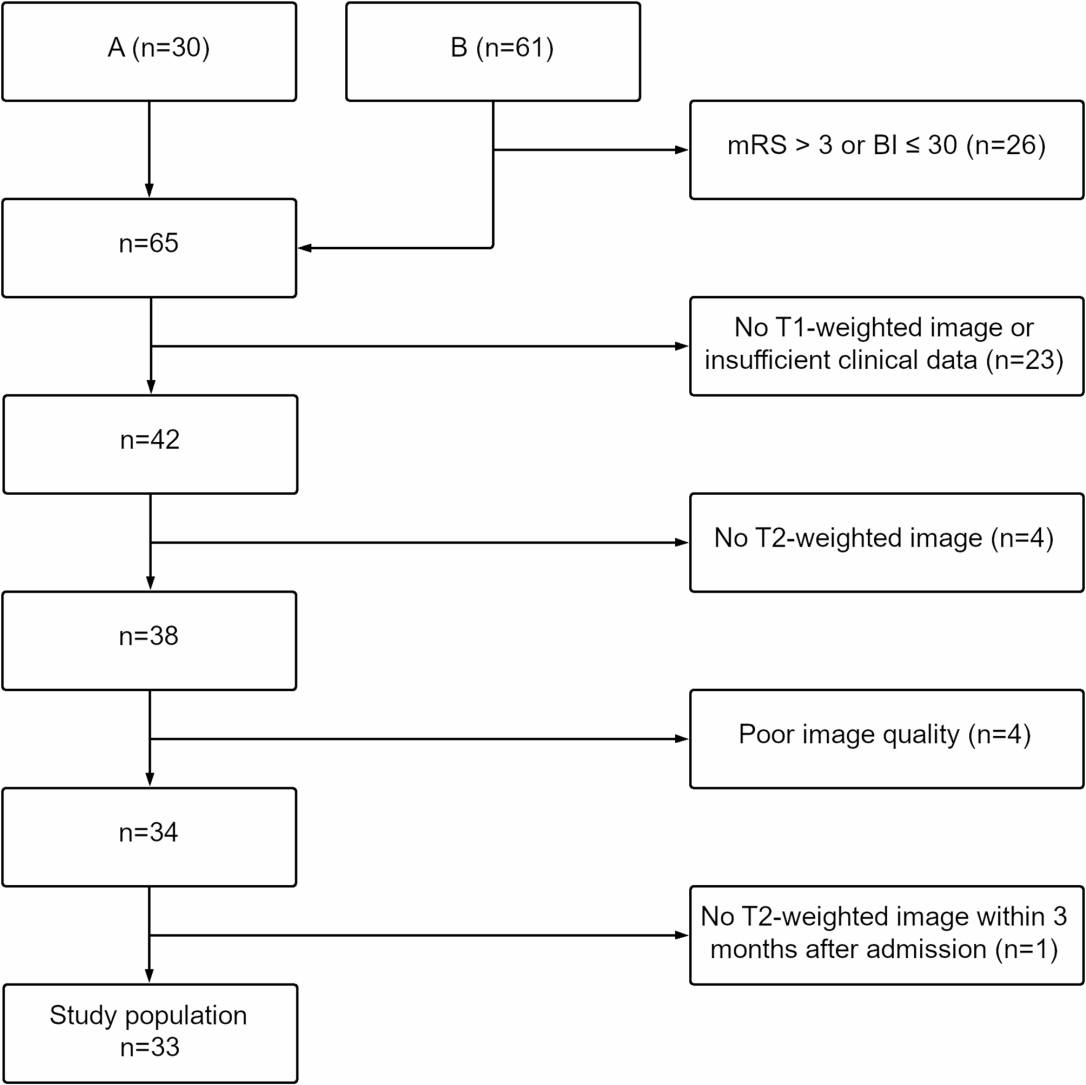
Dataset composition. Flowchart depicting inclusion and exclusion of participants, which are derived from 2 independent cohorts A [22] and B [23]

The data used in this study were obtained by combining two independent cohorts of acute stroke patients whose details have been published previously [22, 23]. All patients were treated at the University Medical Center Hamburg-Eppendorf between 2012 and 2020. Patients of both studies were integrated into one novel cohort comprising 33 patients with initial severe motor deficits. We defined severe motor deficit as an mRS > 3 (moderately severe or severe disability) or a Barthel Index ≤ 30 (severe dependence) at admission, which is following the inclusion criteria of our studies. This approach was used in our previous studies on the same cohort [18, 19, 24, 25]. Please see the Supplementary Methods for details of cohort integration; a flowchart of dataset composition is given in Fig. 1. Patients received a structural brain MRI within 3–14 days after stroke onset (3–14 days in study A and 3–5 days in study B). In three patients, imaging including T1- and T2-weighted data was available later (*n* = 1 within 1 month and *n* = 2 within 3 months). FLAIR data was unavailable in one patient, and T2* data was used for further analysis. The National Institute of Health Stroke Scale (NIHSS) assessed the initial deficit. Initial stroke treatment, such as thrombectomy or thrombolysis, can lead to immediate and significant improvements in patient symptoms. To exclude the effects of such treatment, we used the NIHSS at the time of study inclusion rather than at hospital admission (i.e., after acute treatment). The functional outcome was operationalized by the mRS (see Supplementary Table 1) in the late subacute stage of recovery three months after stroke or six months in four patients where data for the earlier time point were unavailable. This approach also aligns with our previous reports [18, 19, 24, 25]. We have no information on pre-stroke cognitive data of the patients. The original studies were conducted in line with the ethical declaration of Helsinki and were granted permission by the local ethics committee of the Chamber of Physicians Hamburg (PV3777, PV5442, and PV5357). All participants or their legal guardians provided informed consent.

### Image acquisition and processing

**Fig. 2:**
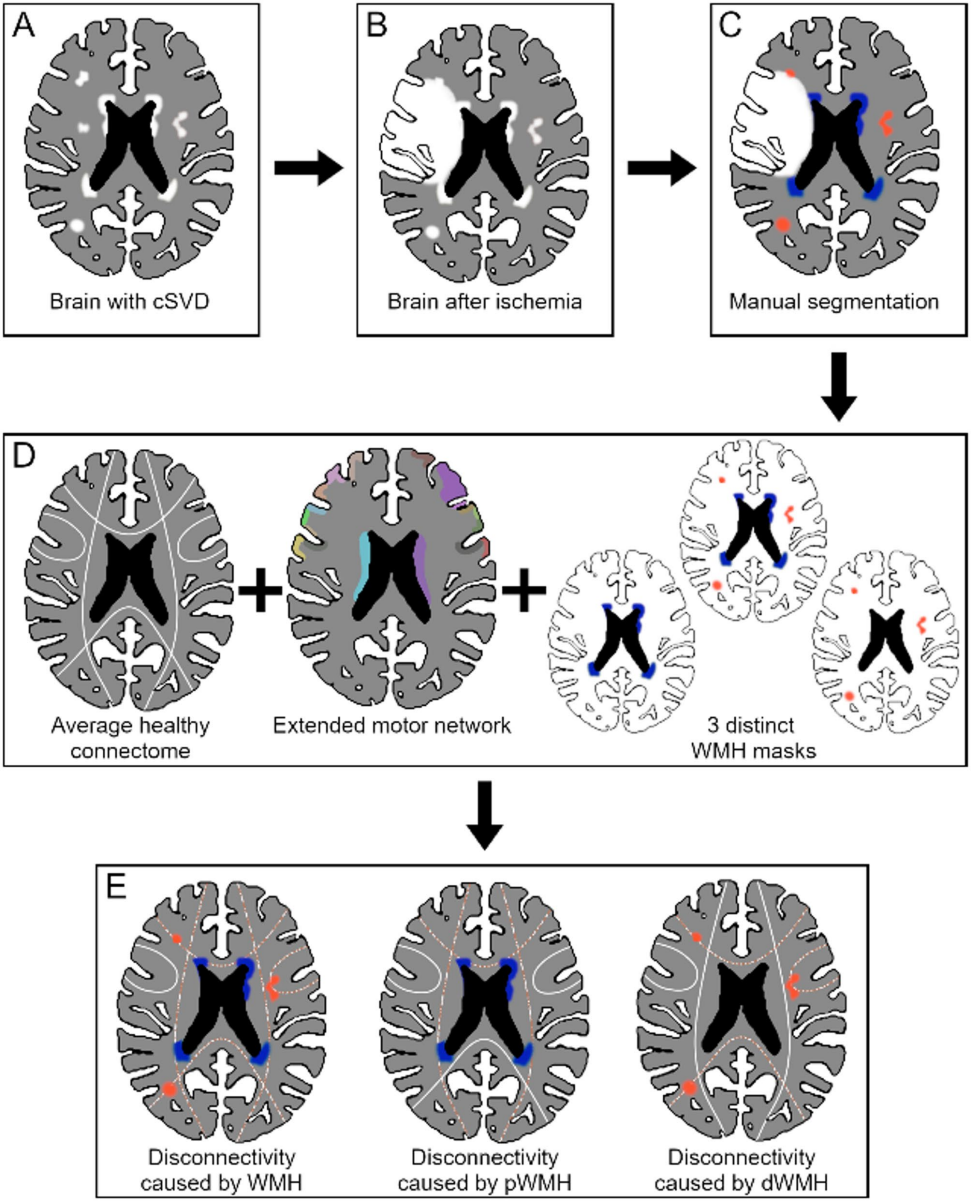
Image processing and disconnectivity measures of WMH-related networks. Overview of image processing and disconnectivity measure acquisition. **A**: Visible WMH appearing in T2-weighted MRI due to cerebral small vessel disease. **B**: Stroke-lesion which overlays the pre-existing WMH. **C**: Manual WMH segmentation. Some are hidden by the stroke lesion and are, therefore, not included in the segmentation. **D**: Segmented WMH are used to create three binary masks: one mask containing all WMH, one with dWMH only, and one with pWMH only. The Network Modification (NeMo) Tool separately processes these three masks with an extended motor network serving as the reference connectome. **E**: The output of the NeMo analyses provides estimates of localized network damage, i.e., disconnection due to WMH, affecting specific cortical and subcortical regions

Imaging was performed with the same 3 Tesla Skyra MRI scanner (Siemens Healthineers, Erlangen, Germany) in all included patients using a 32-channel head coil and a T1-weighted magnetization-prepared rapid gradient echo sequence (repetition time (TR) = 2500 ms, echo time (TE) = 2.12 ms, flip angle = 9°, number of coronal slices = 256, voxel size = 0.8 × 0.8 × 0.9 mm³ and field of view (FOV) = 240 mm) as well as T2-weighted fluid attenuated inverse recovery (FLAIR) datasets (TR = 9000 ms, TE = 86 ms, time to inversion = 2500 ms, flip angle = 150°, number of transversal slices = 43, voxel size = 0.7 × 0.7 × 3.0 mm³ and FOV = 230 mm). WMH and stroke lesions were delineated manually by a trained rater on FLAIR scans using the “Active Contour Segmentation” tool, provided by ITK-SNAP (v3.8.0) [26]. To estimate intra-rater reliability, the segmentation process was repeated in eight randomly selected patients with good results (94.9% for WMH volumes and 98.7% for stroke lesion volumes). Masks of WMH and stroke lesions were normalized to the Montreal Neurological Institute (MNI) 152 standard space using the Advanced Normalization Tools [27]. Normalized WMH masks were divided into pWMH and dWMH by a 10 mm distance threshold to the ventricles [4]. The quality of the image processing steps was carefully assured by repeated visual inspection. The distribution of stroke and WMH lesions is illustrated in Supplementary Fig. 1. Normalized masks of WMH, pWMH, and dWMH were analyzed via the NeMo tool (v2.1a8) [28] (https://kuceyeski-wcm-web.s3.us-east-1.amazonaws.com/upload.html). This tool allows the projection of binary masks in MNI space over a healthy brain network, averaged from whole-brain tractography data of 420 healthy individuals derived from the Human Connectome Project, and calculates the resulting change of connectivity (ChaCo, i.e., loss of connectivity) in a highly reproducible way [28]. As mRS is an outcome measure dominated by stroke deficits in the motor domain [29], an extended motor network atlas was chosen as the output resolution for the NeMo analyses, which encompasses 106 cortical and subcortical brain regions from the brainnetome atlas, defined by their structurally connection to the upper limb representation of the primary motor cortex [30]. An illustration of this individual atlas is given in Supplementary Fig. 2. Figure 2 illustrates the steps of image processing and disconnectivity measure acquisition. ChaCo values, ranging from 0 (not disconnected) to 1 (entirely disconnected), were processed using Python version 3.10 (https://github.com/kjamison/nemo). Disconnectivity values < 0.02 were set to 0, and values > 1 were set to 1, as suggested, since such values can be attributed to noise within the NeMo Tool [25, 28].

### Statistical analysis

**Fig. 3:**
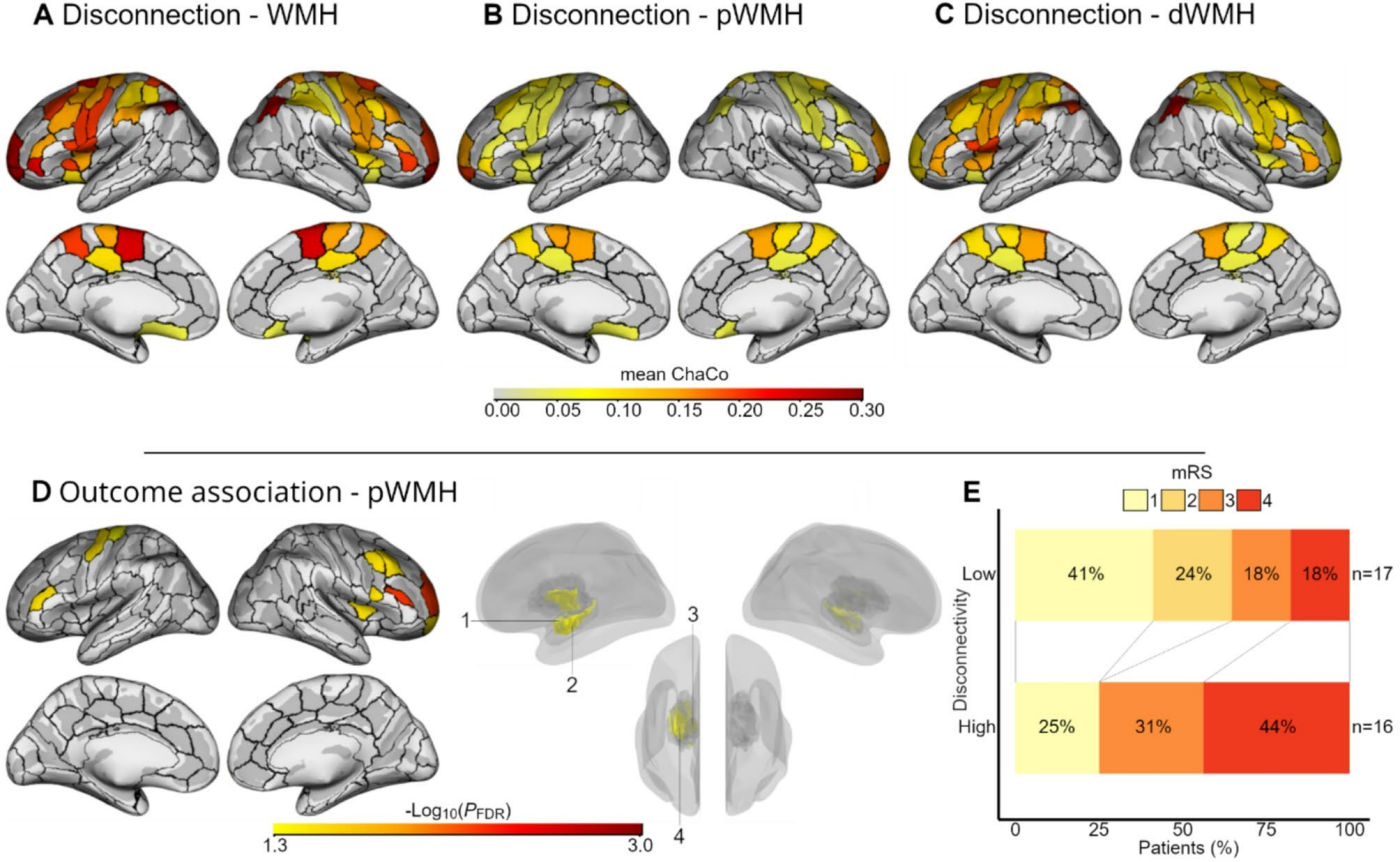
Localized WMH-related network disconnections and correlations with outcome after stroke.** A-C**: Significant region-wise mean ChaCo values, presented separately for total (A), periventricular (B, pWMH), and deep WMH (C, dWMH), visualized as a brain surface overlay. ChaCo value of 1 corresponds to total disconnection from the remaining brain network, and 0 corresponds to no disconnection at all. An unpaired one-tailed *t*-test against 0 was performed to assess the significance of these measures. **D**: Correlation between dichotomized disconnection of brain regions, caused by pWMH, and outcome after stroke with *P*_FDR_-values (-Log_10_ transformed) of associations projected onto the corresponding cortical and subcortical areas. 1: Medial amygdala. 2: Rostral hippocampus. 3: Lateral pre-frontal and pre-motor thalamus. 4: Globus pallidus. **E**: Shift-plot depicting the influence of “high” vs. “low” disconnectivity group on mRS at follow-up, based on mean disconnectivity of all significant regions (*n* = 17, see Table 2). For illustration purposes only

Statistical analysis was performed using R version 4.3.1. Two different statistical analyses were carried out. First, unpaired one-sided *t*-tests were computed to assess which regions were significantly disconnected from the remaining brain network at group level (*P* < 0.05), comparing each region’s mean ChaCo value against 0 (Fig. 3A-C). In addition, a mean ChaCo of subcortical and cortical areas was calculated separately. The difference in these measures between the three groups (total WMH, pWMH, dWMH) was determined using paired two-tailed *t*-tests. Second, independent from the group level disconnection analyses, ordinal logistic regression models (function *polr* from the MASS package [31]) were fitted for mRS at follow-up as the dependent variable (Fig. 3D, E). For this analysis, cortical and subcortical areas exhibiting complete or almost complete connection or disconnection (operationalized by skewness of γ_1_ > 1.3 or γ_1_< -1.3, *n* = 7, or a median of 0 or 1, *n* = 8, respectively) were excluded from these regression analyses. This approach was introduced in our previous report [25]. The final set of regions undergoing outcome regression modeling is given in Supplementary Table 2. Similar to earlier reports on structural biomarkers in stroke [18, 19, 24, 32] or psychiatric disorders [33], we divided the patients’ group by the median into two subsets per region (larger and smaller ChaCo values, i.e., *low* and *high* disconnectivity). The odd number of participants (*n* = 33) resulted in a true median, which we assigned to the “small” disconnectivity group. Global and region-specific WMH-related high/low network disconnectivity was treated as the independent variable of interest. The following nuisance variables were included to adjust target effects: age, NIHSS at study inclusion, stroke lesion volume, and total WMH volume. Stroke lesion and WMH volumes were log_10_-transformed to achieve normal data distribution. Given strong correlations between age and WMH [3], and assuming a priori a correlation between WMH volume and WMH-related network damage, age and total WMH volume were included after residualization against the region-specific WMH-related disconnectivity [17, 19]. Odds ratios (OR), including 95% confidence intervals (CI), and *P* values were extracted from the models. An OR of higher than 1 would indicate a higher probability of scoring one level higher in mRS at follow-up, i.e., having a worse functional outcome for patients in the higher disconnectivity group. To assess the amount of additionally explained variance by the extent of network damage affecting each region (predictor of interest), gain in R² was given compared to the comparative base model without the predictor of interest. The base model statistics are summarized in Supplementary Table 3. Analyses were repeated for each region for WMH (*n* = 91) and the subcomponents dWMH (*n* = 71) and pWMH (*n* = 93) in separate iterations according to Supplementary Table 2, with subsequent *P* value correction for multiple testing using the false discovery rate (FDR) method.

## Results

### Demographic and clinical data

Table 1 summarizes demographic and clinical data of the 33 severely affected first-ever ischemic stroke patients included in this analysis.

**Table 1:** Demographic and clinical data

Clinical characteristic	Total (n = 33)
Age, years (mean, SD)	70.7 (12.2)
Sex	
- Male (%)	16 (48%)
- Female (%)	17 (52%)
Affected hemisphere	
- Left (%)	13 (39%)
- Right (%)	20 (61%)
Handedness	
- Left (%)	2 (6%)
- Right (%)	31 (94%)
Dominant side stroke (%)	13 (39%)
Brainstem stroke (%)	5 (15%)
Initial therapy	
- Thrombolysis only (%)	5 (15%)
- Thrombectomy only (%)	1 (3%)
- Both (%)	13 (39%)
NIHSS at study inclusion	
- Mean (SD)	7.8 (3.6)
- Range	1–17
mRS at follow-up	
- Median (Q1-Q3)	3 (1–4)
- Range	1–4
Stroke volume (ml, mean, SD)	37.9 (50.4)
WMH volume (ml, mean, SD)	15.3 (10.6)
pWMH volume (ml, mean, SD)	9.5 (6.3)
dWMH volume (ml, mean, SD)	5.8 (4.7)

† Abbreviations: mRS, modified Rankin Scale; NIHSS, National Institute of Health Stroke Scale; WMH, White Matter Hyperintensities of presumed vascular origin; pWMH, periventricular WMH; dWMH, deep WMH

### WMH-related network damage after stroke

Out of the 106 regions of the extended motor network, 100 areas were significantly disconnected by total WMH, 100 by pWMH, and 98 by dWMH. A cortical projection of significant mean ChaCo values is given in Fig. 3A-C. Across cortical regions exhibiting significant disconnection, the average ChaCo value was 0.19 ± 0.06 (mean ± SD) for total WMH, 0.09 ± 0.05 for pWMH, and 0.13 ± 0.04 for dWMH. Deep WMH led to higher levels of cortical disconnectivity than pWMH (*t*(67) = 5.2, *P* < 0.001), although dWMH volume was lower than pWMH volume (Table 1, t(32) = -6.6, *P* < 0.001). Across subcortical regions exhibiting significant disconnection, the average ChaCo value was 0.20 ± 0.09 for total WMH, 0.15 ± 0.07 for pWMH, and 0.09 ± 0.06 for dWMH. Periventricular WMH led to higher levels of subcortical disconnectivity than dWMH (*t*(29) = 3.9, *P* < 0.001), following pWMH volume being higher than dWMH volume. Regions exhibiting highest mean disconnectivity (i.e. ChaCo) caused by total WMH were dorsal caudate (0.39 ± 0.16, Mean ± SD), dorsolateral putamen (0.35 ± 0.22), sensory (0.33 ± 0.24) and posterior parietal thalamus (0.31 ± 0.19) as well as lateral area 10 (0.30 ± 0.13) on the contralesional hemisphere. Areas with the highest mean disconnectivity caused by pWMH were contralesional (0.36 ± 0.16) and ipsilesional (0.27 ± 0.20) dorsal caudate, as well as posterior parietal (0.25 ± 0.17) and sensory thalamus (0.25 ± 0.21) on the contralesional hemisphere. The highest mean disconnectivity caused by dWMH affected the contralesional dorsolateral putamen (0.27 ± 0.20) and both the contralesional (0.25 ± 0.22) and ipsilesional (0.25 ± 0.21) rostrodorsal area 39.

### Association between disconnectivity measures and functional outcome

The disconnectivity of multiple regions affected by pWMH was significantly linked to a worse outcome at follow-up, independent of total WMH volume, age, lesion volume, and initial NIHSS. Specifically, on the ipsilesional hemisphere, areas of the middle frontal gyrus contributing to the dorsolateral prefrontal cortex (DLPFC), including ventral Brodmann area 9/46, area 46, ventrolateral area 8, lateral area 10, and inferior frontal junction, additionally explained up to 17.0% of variance. Patients exhibiting higher network disconnectivity in these regions had higher odds of showing higher mRS at follow-up when compared to patients with lower network disconnectivity (Table 2; Fig. 3).

**Table 2:** Localized pWMH-related network damage relates to the outcome after stroke

Region		Hemisphere	*P*_FDR_	OR (95% CI)	*R*^2^-gain
Precentral gyrus	Area 4, upper limb region	Contralesional	0.043	7.41 (1.67–38.96)	6.3%
	Caudal dorsolateral area 6	Contralesional	0.034	9.52 (1.98–58.63)	4.9%
Middle frontal gyrus	Ventral area 9/46	Contralesional	0.039	8.42 (1.78–49.79)	6.8%
		Ipsilesional	0.005	72.32 (6.51-1,542.21)	17.0%
	Area 46	Ipsilesional	0.005	72.32 (6.51-1,542.21)	17.0%
	Ventrolateral area 8	Ipsilesional	0.034	14.07 (2.20-126.13)	8.0%
	Inferior frontal junction	Ipsilesional	0.034	14.07 (2.20-126.13)	8.0%
	Lateral area 10	Ipsilesional	0.034	11.78 (2.18–90.58)	10.3%
Inferior frontal gyrus	Ventral area 44	Ipsilesional	0.034	14.07 (2.20-126.13)	8.0%
	Inferior frontal sulcus	Contralesional	0.034	10.39 (2.14–64.57)	9.6%
		Ipsilesional	0.005	72.32 (6.51-1,542.21)	17.0%
Insular gyrus	Dorsal dysgranular insula	Ipsilesional	0.034	14.07 (2.20-126.13)	8.0%
Amygdala	Medial amygdala	Contralesional	0.039	7.36 (1.71–36.83)	6.5%
Basal ganglia	Globus pallidus	Contralesional	0.039	7.82 (1.77–40.20)	6.5%
Hippocampus	Rostral hippocampus	Contralesional	0.039	7.82 (1.77–40.20)	6.5%
Thalamus	Lateral pre-frontal thalamus	Contralesional	0.034	8.61 (1.93–46.02)	7.5%
	Pre-motor thalamus	Contralesional	0.034	8.61 (1.93–46.02)	7.5%

† Significant associations between dichotomized pWMH-related network damage affecting cortical and subcortical brain regions and outcome after stroke. Results are adjusted for age, initial NIHSS, lesion, and total WMH volumes. ORs with 95% CIs are given for patients with higher disconnectivity (reference) of rising one level in mRS compared to patients with lower disconnectivity for the specific region. *P* values are FDR corrected (*P*_FDR_) for 93 tests. R^2^-gain is given as additional explained variance (R^2^) compared to R^2^ of the base model (39.5%)

On the inferior frontal gyrus, higher disconnection of ventral area 44, the inferior frontal sulcus, and the insular gyrus were similarly associated with worse outcomes after stroke. On the contralesional hemisphere, associations between pWMH-related network damage and worse outcomes were detected for upper limb area 4 of the primary motor cortex and caudal dorsolateral area 6 of the precentral gyrus, area 9/46, and inferior frontal gyrus of the middle and inferior frontal gyrus, respectively. Finally, multiple contralesional subcortical nuclei, the amygdala, globus pallidus, hippocampus, and thalamus also showed significant associations. Compared to pWMH, localized network disconnectivity of WMH or dWMH did not show similar relationships with outcome after stroke (all *P*_FDR_>0.1).

For sensitivity analyses, we recomputed the base model considering pWMH and dWMH volumes and, from a network perspective, the average ChaCo for WMH, pWMH, and dWMH instead of total WMH volume. In these alternative base models, only pWMH-mediated global network damage significantly contributed to the base model (*P* = 0.025, Supplementary Tables 4 and 5) and improved the variance explanation from 39.5 to 45.4%. Primary outcome models based on the subcomponent of pWMH (*n* = 93) were refit and adjusted for global pWMH network damage instead of WMH volume. Results remained largely stable after using this improved base model (Supplementary Table 6).

## Discussion

The main finding of the present work was that localized pWMH-related network damage, disconnecting distinct brain regions from large-scale brain networks, was linked to a worse outcome after a severe stroke. The association was independent of the initial deficit, age, the overall volume of WMH, and the stroke lesion. Specifically, we showed that the disconnection of ipsilesional DLPFC, insular cortex, contralesional primary and secondary motor areas, and subcortical nuclei, including the thalamus, from large-scale networks by pWMH, exerted a negative influence on stroke recovery. This study contributes to the emerging field of research on WMH-related network effects, in addition to WMH burden as a general risk factor for worse stroke outcomes, to understand stroke outcome variability. WMH burden has repeatedly been associated with worse outcomes after stroke in clinical populations [6, 8]. However, in patients with severe deficits caused by large vessel occlusion, previous studies have questioned such observations as they did not find a clear relationship between WMH and revascularizing treatment gains and outcomes [34, 35]. In agreement with this data, we did not detect significant correlations between the established WMH volume and mRS at follow-up in our cohort, neither for total WMH nor for pWMH and dWMH (Supplementary Tables 3 and 4). In contrast, we found that the degree of network disconnectivity affecting specific cortical and subcortical brain regions associated with pWMH was correlated with functional outcome. Interestingly, already substituting the WMH volume in the base model by pWMH-related global network damage explained an additional amount of variance in mRS at follow-up, which highlights that WMH-related network damage seems to be particularly informative for outcome inference after severe stroke. Data on WMH-related network effects are still limited. Just recently, a first study found that their integration could improve cross-sectional inference of non-verbal cognitive deficits in aphasic stroke patients [20].

We found only pWMH-related global and localized network damage linked to the clinical outcome, but not network damage derived from total WMH or dWMH. A similar observation was made for recovery after primary intracerebral hemorrhage, where higher pWMH, but not dWMH volumes, were associated with an unfavorable long-term outcome [36]. Another study could link the extent of cortical atrophy to pWMH-related disruption of connecting white matter tracts. This association was not evident for total WMH or dWMH-affected networks [37]. There are different possible explanations for these differences. On the one hand, brain regions involved in recovery processes may be connected by fibers running through areas typically affected by pWMH. On the other hand, we speculate statistically that the relatively stable spatial distribution of pWMH could result in more robust effects at the group level in smaller cohorts than dWMH.

The region-specific assessment of network damage related to pWMH allowed us to determine whether localized network disconnection affecting specific cortical and subcortical regions might impact clinical outcomes. Four regions will be discussed below.

On the ipsilesional hemisphere, higher disconnection of areas contributing to the DLPFC was correlated with a worse outcome. Instead of short association fibers, whose disruption would expectedly be driven mainly by dWMH [37], pWMH might act on the DLPFC by disrupting thalamic association fibers [38]. This is further supported by the concomitant significant disconnection of thalamic brain regions (Table 2; Fig. 3) and findings of a previous study showing that WMH are, to a large extent, located within the anterior thalamic radiatio and superior longitudinal fasciculus [39], which both connect to the DLPFC. Previous studies have shown that non-invasive stimulation of the DLPFC might serve as one innovative treatment approach to enhance functional recovery after stroke, particularly in the cognitive domain [40]. Herein, stimulation effects were more likely to affect memory, attentional, and executive [41] than motor functions [42]. One study found that an increased functional connectivity between the contralesional DLPFC and the mid-ventrolateral prefrontal cortex was linked to better hand function in chronic stroke patients [43]. Vice versa, others reported that subacute stroke patients, in whome ipsilesional DLPFC areas gradually disconnected from larger connectomes by the lesion, were at higher risks for impaired natural recovery [13]. The present study furthers these insights and shows that DLPFC disconnection from brain networks, potentially also caused by pre-existing pWMH, can increase, at least partly, the risk for an unfavorable outcome.

A second significant association for pWMH-related network damage and outcome was found for the contralesional precentral gyrus, including the primary motor cortex and areas contributing to the dorsolateral and ventral premotor cortex. Given the associations between pWMH-related disconnectivity and cortical atrophy previously described [37], we noticed an interesting parallel comparing the current results to an earlier report from our group. Based on a similar cohort of severely impaired stroke patients, we found that reduced cortical thickness of the contralesional precentral gyrus at baseline was significantly associated with a worse outcome [18]. Hence, the relationship between clinical outcome and cortical thickness might be driven by pWMH-related network disturbances over time. Combined analyses of baseline cortical anatomy and WMH disconnections are warranted to systematically explore the interrelationship between both measures and their importance for recovery, following the emerging concepts of structural brain reserve after stroke [17].

Finally, the dorsal insula on the ipsilesional side and subcortical nuclei on the contralesional side, including the premotor and lateral prefrontal thalamus, were identified as risk factors for a poorer outcome when disconnected from larger brain networks by pWMH. These findings are in line with prior research. For instance, lesions within the insular lobe have been associated with more significant initial deficits [44], worse outcomes [45], and higher mortality [46]. For the thalamus, previous reports have linked ipsilesional thalamic volume and secondary thalamic atrophy after stroke to worse sensorimotor [47] and cognitive performance [48]. WMH-related network damage affecting the insula or thalamus might make such highly interconnected hubs particularly susceptible to further, and then, critical disconnection caused by the stroke lesion itself. Threatened by disconnection and secondary atrophy, key nodes of the human motor system, such as the thalamus, could lose essential regulatory functions necessary for regular movements [49] or, as animal studies have shown, re-learning processes [50].

### Limitations

There are several important limitations worth noting. First, analyses were conducted on a small sample of severely impaired acute stroke patients. Recruiting severely impaired stroke patients for cranial MR imaging in the acute phase is challenging, as many of these patients are clinically unstable and unable to provide written consent. Additionally, many participants had to be excluded due to motion artefacts and, subsequently, poor image quality, as is often the case in such severely impaired patients. To alleviate effects of this small sample size, statistical modeling was corrected for multiple testing. However, the sensitivity and generalizability of the present analyses are likely to be reduced, and the presented OR must be interpreted with care. Therefore, prospective studies are needed to reaffirm our findings. On the same vein, we are aware of the high number of variables in relation to the small cohort size and recognize that this might lower the robustness of the multivariable models. However, we believe it would be illogical to omit any of these variables, which include age, NIHSS at study inclusion, stroke lesion volume and total WMH volume, given that they are recognized as crucial factors in stroke recovery [6, 7, 8]. Second, multivariate ordinal logistic regression models were computed for outcome inference, in line with our earlier work [18, 19, 25]. Alternative advanced approaches, including ridge and partial least squares regressions, might be reasonable alternatives when considering collinearity of independent predictors or spatial autocorrelations of neighboring regions. Our approach of linear residualization of age and WMH against the region-specific WMH-related disconnectivity [17, 19] provides remedy for the former limitation. Third, brain imaging for acute lesion volume and WMH segmentation, based on T1- and T2-weighted MRI, was conducted mainly within the first two weeks after stroke, but up to three months after stroke in two patients. Hence, atrophy processes influencing lesion topography and segmentation, image normalization, and altering WMH with a slight risk for additional de novo manifestations of WMH after stroke may affect the present modeling results. For further sensitivity analyses, we repeated all analyses while excluding three patients whose imaging was later than two weeks. Most significant regions remained stable (Supplementary Table 7). This result, however, must be interpreted with caution given the low precision of the OR estimates. In this context, the factor *follow-up time point* (three or six months after stroke) could also influence our findings. However, including this factor did not alter our main findings, which aligns with our earlier work on this cohort [14]. Fourth, WMH located within the primary stroke lesion could not be adequately delineated, their extent remained elusive, and they were not included in the binary WMH masks. Also, WMH volumes were underestimated with a bias towards patients with larger stroke lesions. We decided not to address this problem, e.g., by mirroring WMH distributions from the contralesional to the ipsilesional hemisphere to estimate a *pre-stroke* state of the lesioned brain, as such an approach is neither straightforward nor free of further limitations. Fifth, the connectome analysis was based on an arbitrary value of complete streamline disruption evoked by all individual WMH lesions. Whether a more fine-grained definition of the disruptive impact of each WMH lesion, e.g., based on quantitative T2-weighted data or diffusion-based microstructural information, might alter the present findings remains an interesting research question for future multimodal work. Sixth, mRS was used as the outcome measure, a clinical score of global disability dominated by preserved functions of the motor domain. Therefore, the reference network for connectome analyses was biased towards an extended motor network [30]. Alternative outcome measures in the cognitive or language domains might call for alternative reference networks and alter the present results.

## Conclusions

This study shows that pWMH disconnects specific brain regions, including primary and premotor cortices, dorsolateral prefrontal cortices, the insula, and various subcortical nuclei, such as the thalamus, from larger brain networks. Severely impaired stroke patients with a higher amount of disconnection in these key areas of cognitive, learning, and motor functioning are at an increased risk of a worse outcome, independent of the initial deficits, age, lesion volume, and total WMH volume. Hence, this work adds to the emerging concept of WMH-related network disconnections as an essential factor for outcomes and recovery trajectories after stroke. Regarding clinical applications, it may lead to interesting new research questions. For instance, could patients with network-critical WMH distributions benefit from intensive adjustment of cardiovascular risk factors more than patients with less critical network damage? Or could WMH-related network damage with consecutive disruption of cognitively important brain regions help to stratify patients towards motor and cognitive neurorehabilitative training or non-invasive brain stimulation of non-motor areas to enhance recovery after stroke?

## Supplementary Information

Below is the link to the electronic supplementary material.

Supplementary Material 1

## Data Availability

Processed data supporting this study’s findings are available from the authors upon reasonable request. Restrictions might apply to the availability of these data, which were obtained in accordance with the requirements of the local ethics committee. The code supporting the findings is also available from the authors upon request.
