# Supplementary material for: Localized network damage related to white matter hyperintensities is linked to worse outcome after severe stroke
Source: Neurol Res Pract. 2025 Aug 19;7(1):57. doi: 10.1186/s42466-025-00416-w (PMC12366146; doi:10.1186/s42466-025-00416-w)
Supplement: Supplementary file 1 — Supplementary Material 1 [file 42466_2025_416_MOESM1_ESM.docx]

**SUPPLEMENTAL MATERIAL**

**Localized network damage related to white matter hyperintensities is linked to worse outcome after severe stroke.**

Olszówka, Samuel C ^a,†^, Frey, Benedikt M ^a,†^, Feldheim, Jan F ^a^, Frontzkowski, Lukas ^a^, Wróbel, Paweł P ^a^, Backhaus, Winifried ^a^, Higgen, Focko L ^a^, Braaß, Hanna ^a^, Wolf, Silke ^a^, Choe, Chi-un ^a^, Bönstrup, Marlene ^a,b^, Cheng, Bastian ^a^, Thomalla, Götz ^a^, Koch, Philipp J ^c^, Quandt, Fanny ^a^, Gerloff, Christian ^a^, Schulz, Robert ^a^

^a^ University Medical Center Hamburg-Eppendorf, Department of Neurology, Hamburg, Germany

^b^ University Medical Center Frankfurt, Department of Neurology, Frankfurt, Germany

^c^ Department of Neurology, Charite University Medical Center Berlin, Berlin, Germany

^†^ These authors contributed equally to this work

**Supplementary Methods**

**Cohort integration**

For cohort integration, inclusion criteria from study A were retrospectively applied for study B, in line with previous reports [1, 4-6], that were modified Rankin Scale (mRS) > 3 (moderately severe or severe disability, see Supplementary Table 1) or a Barthel Index (BI) ≤ 30 (severe dependance) at admission, age ≥ 18 years, first-ever ischemic stroke with persistent motor deficit of the upper limb and no prior history of severe psychiatric or neurological disorders. After excluding incomplete (n=28) or qualitatively insufficient MRI images (n=4), the final sample size comprised 33 datasets. All 33 of these patients qualified via initial mRS. Out of those, 12 also qualified via initial BI. In one patient, data for initial BI was lacking.

**Supplementary Figures**


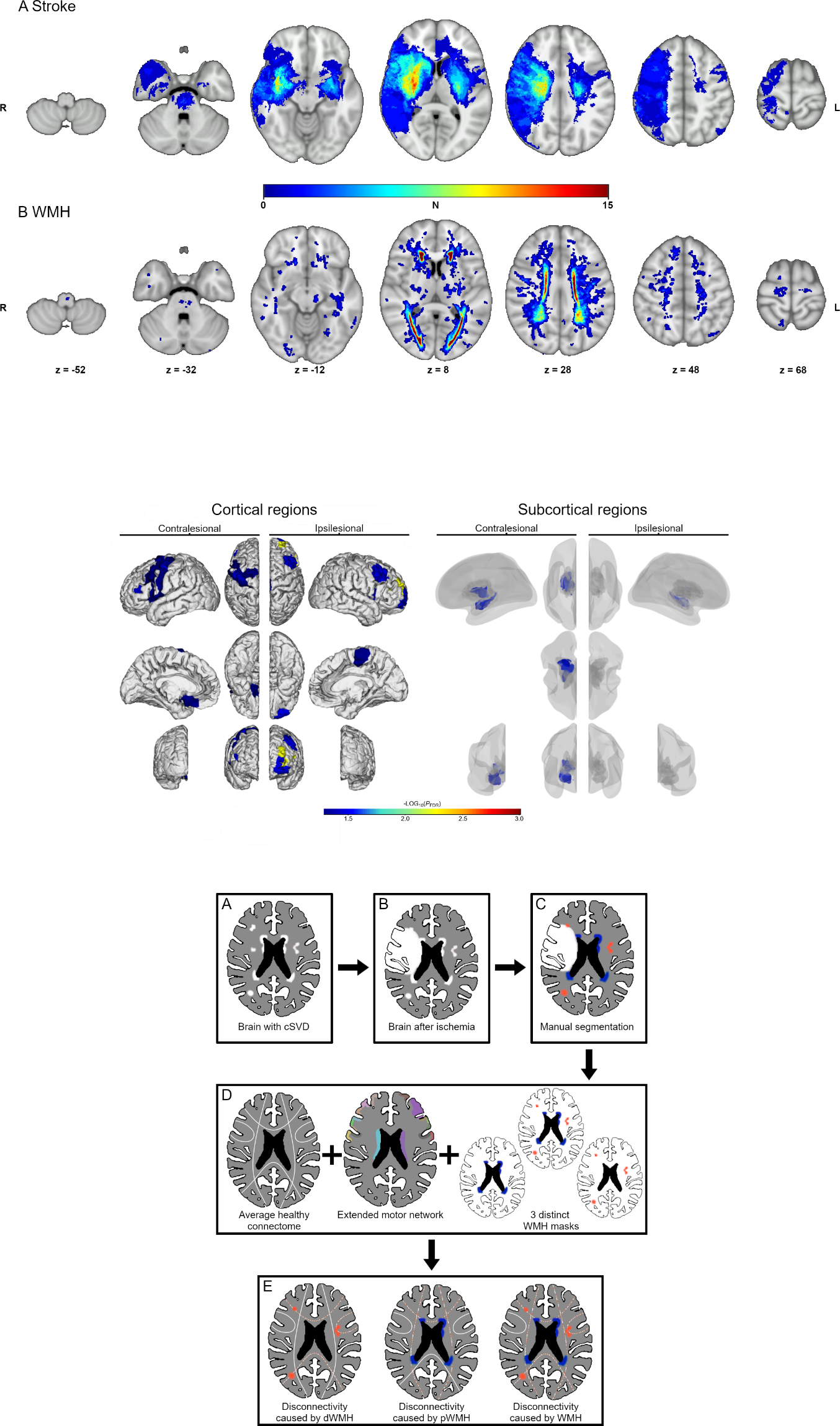


**Supplementary Figure 1. Distribution of stroke and WMH lesions.**

Lesion distribution heatmap for stroke lesions (A) and WMH (B), superimposed on a T1-weighted brain image in MNI standard space. The color represents the number (N) of patients having lesions within an area. The letters “R” and “L” indicate the side of the brain. Z-values indicate the horizontal slice in MNI standard space.


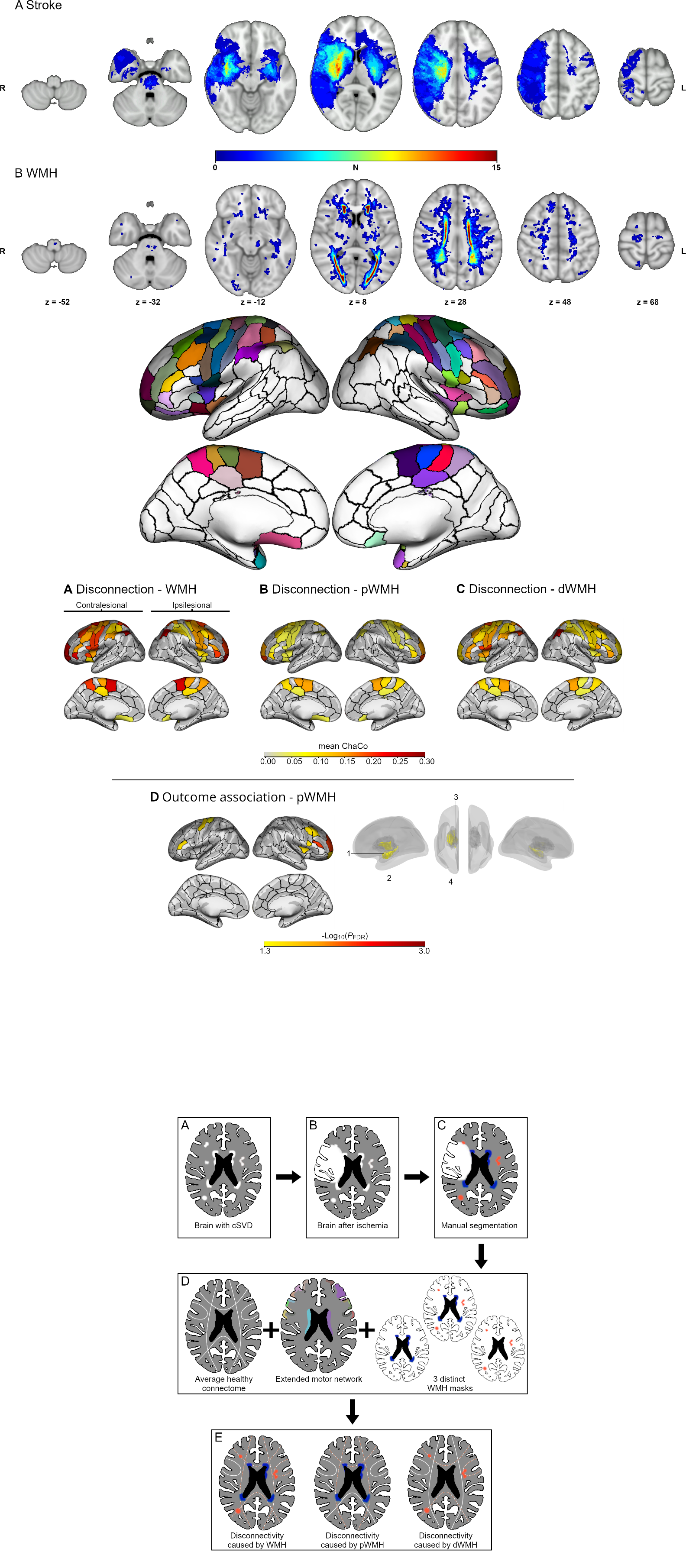


**Supplementary Figure 2. Extended Core Motor Network (cortical regions only).**The cortical regions of the extended core motor network are visualized as a brain surface.

**Supplementary** **Tables**

| Level |  | Description |
| --- | --- | --- |
| 0 |  | No symptoms at all |
| 1 |  | No significant disability despite symptoms: able to carry out all usual duties and activities |
| 2 |  | Slight disability: unable to carry out all previous activities but able to look after own affairs without assistance |
| 3 |  | Moderate disability: requiring some help, but able to walk without assistance |
| 4 |  | Moderately severe disability: unable to walk without assistance, and unable to attend to own bodily needs without assistance |
| 5 |  | Severe disability: bedridden, incontinent, and requiring constant nursing care and attention |

**Supplementary Table 1.** **Modified Rankin Scale**

Modified Rankin Scale (mRS), used to measure the degree of disability in stroke patients [7].

| **Total WMH (WMH)** | | | **Periventricular WMH (pWMH)** | | | **Deep WMH (dWMH)** | |
| --- | --- | --- | --- | --- | --- | --- | --- |
| Contralesional | Ipsilesional | | Contralesional | | Ipsilesional | Contralesional | Ipsilesional |
| A1.2.3ll | | A1.2.3ll | A1.2.3ll | A1.2.3ll | | A1.2.3tru | A1.2.3tru |
| A1.2.3tru | | A1.2.3tru | A1.2.3tru | A1.2.3tru | | A1.2.3ulhf | A1.2.3ulhf |
| A1.2.3ulhf | | A1.2.3ulhf | A1.2.3ulhf | - | | A12.47o | - |
| A10l | | A10l | A10l | A10l | | - | A23c |
| A12.47o | | A12.47o | A12.47o | A12.47o | | A39rd | A39rd |
| - | | A13 | A13 | A13 | | A40rd | A40rd |
| - | | A2 | A2 | - | | A44v | A44v |
| A23c | | A23c | A23c | A23c | | A45r | A45r |
| A39rd | | A39rd | A39rd | A39rd | | - | A46 |
| A40rd | | A40rd | A40rd | A40rd | | A4hf | - |
| A44v | | A44v | A44v | A44v | | A4ll | A4ll |
| A45r | | A45r | A45r | A45r | | A4t | A4t |
| A46 | | A46 | A46 | A46 | | A4tl | - |
| A4hf | | A4hf | A4hf | A4hf | | A4ul | A4ul |
| A4ll | | A4ll | A4ll | A4ll | | A5m | - |
| A4t | | A4t | A4t | A4t | | - | A6cdl |
| A4tl | | - | A4tl | A4tl | | A6cvl | A6cvl |
| A4ul | | A4ul | A4ul | A4ul | | A6dl | A6dl |
| A5m | | A5m | A5l | - | | A6m | A6m |
| A6cdl | | A6cdl | A5m | A5m | | A6vl | - |
| A6cvl | | A6cvl | A6cdl | A6cdl | | A7pc | - |
| A6dl | | A6dl | A6cvl | A6cvl | | A7r | A7r |
| A6m | | A6m | A6dl | A6dl | | A8vl | A8vl |
| A6vl | | A6vl | A6m | A6m | | - | A9.46v |
| A7pc | | A7pc | A6vl | A6vl | | dCa | dCa |
| A7r | | A7r | A7pc | A7pc | | dIa | dIa |
| A8vl | | A8vl | A7r | A7r | | dId | dId |
| A9.46v | | A9.46v | A8vl | A8vl | | dlPu | dlPu |
| - | | cHipp | A9.46v | A9.46v | | GP | GP |
| dCa | | dCa | - | cHipp | | IFJ | - |
| dIa | | dIa | dCa | dCa | | IFS | IFS |
| dId | | dId | dIa | dIa | | lPFtha | lPFtha |
| dlPu | | dlPu | dId | dId | | mAmyg | - |
| GP | | GP | dlPu | dlPu | | mPFtha | mPFtha |
| IFJ | | - | GP | GP | | mPMtha | mPMtha |
| IFS | | IFS | IFJ | IFJ | | NAC | - |
| lPFtha | | lPFtha | IFS | IFS | | - | Otha |
| mAmyg | | mAmyg | lPFtha | lPFtha | | PPtha | PPtha |
| - | | mPFtha | mAmyg | - | | rHipp | - |
| mPMtha | | mPMtha | - | mPFtha | | Stha | Stha |
| NAC | | NAC | mPMtha | mPMtha | | vCa | - |
| Otha | | Otha | NAC | NAC | | - | vIa |
| PPtha | | PPtha | Otha | Otha | | vId.vIg | vId.vIg |
| rHipp | | rHipp | PPtha | PPtha | | vmPu | vmPu |
| Stha | | Stha | rHipp | rHipp | |  |  |
| vCa | | vCa | Stha | Stha | |  |  |
| - | | vIa | vCa | vCa | |  |  |
| vId.vIg | | vId.vIg | - | vIa | |  |  |
| vmPu | | vmPu | vId.vIg | vId.vIg | |  |  |
|  | |  | vmPu | vmPu | |  |  |

**Supplementary Table 2.** **Final list of regions undergoing statistical modeling for each of the three iterations.**

Comprehensive list of all regions undergoing statistical modeling for the three test regimens. After excluding regions with skewed ChaCo distribution (γ_1_ > 1.3 or < -1.3) or median of 0 or 1 [4], respectively, 91 areas were included for the analysis of total WMH, 93 for periventricular WMH, and 71 for deep WMH.

| **Predictor (model A)** | **OR (95% CI)** | ***P*** |
| --- | --- | --- |
| NIHSS at admission | 1.53 (1.16-2.02) | <0.001 |
| Age | 1.04 (0.99-1.10) | 0.140 |
| Log_10_(stroke lesion volume) | 0.99 (0.62-1.59) | 0.978 |
| Log_10_(WMH volume) | 1.88 (0.70-5.08) | 0.202 |

**Supplementary Table 3. Base model details.**

Predictor of interest was mRS at follow up. Stroke lesion volume and WMH volume were Log_10_-transformed to account for skewed distribution. The model was able to explain 39.5% of variance (R^2^).

| **Predictor** | **OR (95% CI)** | ***P*** | **Predictor** | **OR (95% CI)** | ***P*** |
| --- | --- | --- | --- | --- | --- |
| NIHSS at admission | 1.55 (1.19-2.12) | <0.001 | NIHSS at admission | 1.52 (1.18-2.06) | <0.001 |
| Age | 1.03 (0.98-1.09) | 0.202 | Age | 1.05 (0.99-1.11) | 0.097 |
| Log_10_(stroke lesion volume) | 0.99 (0.60-1.60) | 0.966 | Log_10_(stroke lesion volume) | 1.00 (0.62-1.60) | 0.995 |
| Log_10_(pWMH volume) | 2.51 (0.86-8.30) | 0.093 | Log_10_(dWMH volume) | 1.34 (0.65-2.85) | 0.425 |

**Supplementary Table 4. Effect of pWMH and dWMH volume on outcome.**

Predictor of interest was mRS at follow up. Stroke lesion volume and pWMH volume were Log_10_-transformed to account for skewed distribution. Models were able to explain 41.6% and 37.6% of variance (R^2^) for pWMH and dWMH, respectively.

| **Predictor** | ***P*** | **Predictor** | ***P*** | **Predictor** | ***P*** |
| --- | --- | --- | --- | --- | --- |
| NIHSS at admission | 0.002 | NIHSS at admission | 0.002 | NIHSS at admission | 0.002 |
| Age | 0.155 | Age | 0.288 | Age | 0.136 |
| Log_10_(stroke lesion volume) | 0.870 | Log_10_(stroke lesion volume) | 0.745 | Log_10_(stroke lesion volume) | 0.915 |
| Mean WMH ChaCo | 0.225 | Mean pWMH ChaCo | 0.025 | Mean dWMH ChaCo | 0.345 |

**Supplementary Table 5. Effect of total WMH, pWMH and dWMH mediated mean change of connectivity (ChaCo) on outcome.**

Predictor of interest was mRS at follow up. Stroke lesion volume was Log_10_-transformed to account for skewed distribution. Models were able to explain 39.2%, 45.4% and 38.1% of variance (R^2^) for each of total WMH, pWMH and dWMH, respectively.

| Region |  | Hemisphere | *P*_FDR_ | OR (95% CI) | R^2^-gain |
| --- | --- | --- | --- | --- | --- |
| Precentral gyrus | Area 4, upper limb region | Contralesional | 0.038 | 8.45 (1.85-46.13) | 1.9% |
|  | Caudal dorsolateral area 6 | Contralesional | 0.038 | 9.49 (2.02-54.44) | 2.5% |
| Middle frontal gyrus | Ventral area 9/46 | Contralesional | 0.038 | 8.84 (1.87-51.29) | 1.9% |
|  |  | Ipsilesional | 0.011 | 50.66 (5.07-1013.33) | 9.9% |
|  | Area 46 | Ipsilesional | 0.011 | 50.66 (5.07-1013.33) | 9.9% |
|  | Ventrolateral area 8 | Ipsilesional | 0.041 | 10.27 (1.80-79.17) | 1.8% |
|  | Inferior frontal junction | Ipsilesional | 0.041 | 10.27 (1.80-79.17) | 1.8% |
|  | Lateral area 10 | Ipsilesional | 0.038 | 13.47 (2.48-104.21) | 6.5% |
| Inferior frontal gyrus | Ventral area 44 | Ipsilesional | 0.041 | 10.27 (1.80-79.17) | 1.8% |
|  | Inferior frontal sulcus | Contralesional | 0.038 | 10.50 (2.20-62.21) | 3.7% |
|  |  | Ipsilesional | 0.011 | 50.66 (5.07-1013.33) | 9.9% |
| Insular gyrus | Dorsal dysgranular insula | Ipsilesional | 0.041 | 10.27 (1.80-79.17) | 1.8% |
| Amygdala | Medial amygdala | Contralesional | 0.038 | 8.44 (1.90-44.24) | 2.3% |
| Basal ganglia | Globus pallidus | Contralesional | 0.038 | 8.45 (1.86-45.05) | 1.6% |
| Hippocampus | Rostral hippocampus | Contralesional | 0.038 | 8.45 (1.86-45.05) | 1.6% |
| Thalamus | Lateral pre-frontal thalamus | Contralesional | 0.038 | 9.73 (2.11-53.81) | 2.6% |
|  | Pre-motor thalamus | Contralesional | 0.038 | 9.73 (2.11-53.81) | 2.6% |

**Supplementary Table 6. Sensitivity analysis where the model was adjusted for pWMH-mediated global network damage instead of WMH volume.**

Sensitivity analysis for significant associations between dichotomized pWMH-related network damage affecting cortical and subcortical brain regions and outcome after stroke, where the model was adjusted for pWMH-mediated global network damage (i.e., mean ChaCo within the extended motor network) instead of WMH volume. Thus, results are adjusted for age, initial NIHSS, lesion, and pWMH-mediated global network damage. Model details are given in Supplementary Table 4. ORs with 95% CIs are provided for patients with higher disconnectivity (reference) of rising one level in mRS compared to patients with lower disconnectivity for the specific region. *P* values are FDR corrected (*P*_FDR_) for 93 tests. R^2^-gain is given as additional explained variance (R^2^) compared to R^2^ of the modified base model (45.4%).

| Region | | Hemisphere | *P* | OR (95% CI) | R^2^-gain |
| --- | --- | --- | --- | --- | --- |
| Precentral gyrus | Area 4 (upper limb region) | Contralesional | 0.001 | 7.25 (1.53-41.53) | 6.5% |
|  | Caudal dorsolateral area 6 | Contralesional | 0.001 | 7.73 (1.51-49.21) | 5.7% |
| Middle frontal gyrus | Ventral area 9/46 | Ipsilesional | 0.001 | 32.13 (3.61-517.02) | 12.8% |
|  |  | Contralesional | 0.053 | 4.38 (0.98-22.21) | 3.7% |
|  | Area 46 | Ipsilesional | 0.001 | 32.13 (3.61-517.02) | 12.8% |
|  | Ventrolateral area 8 | Ipsilesional | 0.034 | 6.21 (1.15-39.69) | 3.4% |
|  | Inferior frontal junction | Ipsilesional | 0.034 | 6.21 (1.15-39.69) | 3.4% |
|  | Lateral area 10 | Ipsilesional | 0.014 | 7.37 (1.49-44.80) | 7.2% |
| Inferior frontal gyrus | Ventral area 44 | Ipsilesional | 0.034 | 6.21 (1.15-39.69) | 3.4% |
|  | Inferior frontal sulcus | Ipsilesional | 0.001 | 32.13 (3.61-517.02) | 12.8% |
|  |  | Contralesional | 0.011 | 7.74 (1.57-46.76) | 7.5% |
| Insular gyrus | Dorsal dysgranular insula | Ipsilesional | 0.050 | 5.33 (1.00-33.19) | 3.1% |
| Amygdala | Medial amygdala | Contralesional | 0.047 | 4.63 (1.02-23.22) | 2.9% |
| Basal ganglia | Globus pallidus | Contralesional | 0.038 | 5.11 (1.09-27.02) | 2.9% |
| Hippocampus | Rostral hippocampus | Contralesional | 0.038 | 5.11 (1.09-27.02) | 2.9% |
| Thalamus | Lateral pre-frontal thalamus | Contralesional | 0.008 | 10.86 (1.83-89.11) | 8.0% |
|  | Pre-motor thalamus | Contralesional | 0.008 | 10.86 (1.83-89.11) | 8.0% |

**Supplementary Table 7. Sensitivity analysis where patients with imagery not taken immediately after stroke onset were excluded.**

Sensitivity analysis for significant associations between dichotomized pWMH-related network damage affecting cortical and subcortical brain regions and outcome after stroke, where patients with imagery not obtained immediately after stroke onset (n=3) were excluded. Results are adjusted for age, initial NIHSS, lesion, and total WMH volumes. ORs with 95% CIs are given for patients with higher disconnectivity (reference) of rising one level in mRS compared to patients with lower disconnectivity for the specific region. R^2^-gain is provided as additional explained variance (R^2^) compared to R^2^ of the base model (39.5%).

**References**

1. Asmussen L, Frey BM, Frontzkowski LK, Wrobel PP, Grigutsch LS, Choe CU, Bonstrup M, Cheng B, Thomalla G, Quandt F, Gerloff C, Schulz R (2024) Dopaminergic mesolimbic structural reserve is positively linked to better outcome after severe stroke. Brain Commun 6:fcae122

2. Backhaus W, Braass H, Higgen FL, Gerloff C, Schulz R (2021) Early parietofrontal network upregulation relates to future persistent deficits after severe stroke-a prospective cohort study. Brain Commun 3:fcab097

3. Bonstrup M, Krawinkel L, Schulz R, Cheng B, Feldheim J, Thomalla G, Cohen LG, Gerloff C (2019) Low-Frequency Brain Oscillations Track Motor Recovery in Human Stroke. Ann Neurol 86:853-865

4. Frontzkowski L, Fehring F, Frey BM, Wrobel PP, Reibelt A, Higgen F, Wolf S, Backhaus W, Braass H, Koch PJ, Choe CU, Bonstrup M, Cheng B, Thomalla G, Gerloff C, Quandt F, Schulz R (2024) Frontoparietal Structural Network Disconnections Correlate With Outcome After a Severe Stroke. Hum Brain Mapp 45:e70060

5. Rojas Albert A, Backhaus W, Graterol Perez JA, Braabeta H, Schon G, Choe CU, Feldheim J, Bonstrup M, Cheng B, Thomalla G, Gerloff C, Schulz R (2022) Cortical thickness of contralesional cortices positively relates to future outcome after severe stroke. Cereb Cortex 32:5622-5627

6. Sadeghihassanabadi F, Frey BM, Backhaus W, Choe CU, Zittel S, Schon G, Bonstrup M, Cheng B, Thomalla G, Gerloff C, Schulz R (2022) Structural cerebellar reserve positively influences outcome after severe stroke. Brain Commun 4:fcac203

7. van Swieten JC, Koudstaal PJ, Visser MC, Schouten HJ, van Gijn J (1988) Interobserver agreement for the assessment of handicap in stroke patients. Stroke 19:604-607
